# Relativistic inverse Compton scattering of photons from the early universe

**DOI:** 10.1038/s41598-017-17104-8

**Published:** 2017-12-05

**Authors:** Siddharth Malu, Abhirup Datta, Sergio Colafrancesco, Paolo Marchegiani, Ravi Subrahmanyan, D. Narasimha, Mark H. Wieringa

**Affiliations:** 1 Centre of Astronomy, Indian Institute of Technology Indore, Simrol, Khandwa Road, Indore, 453552 India; 2 School of Physics, University of the Witwatersrand, Private Bag 3, WITS-2050 Johannesburg, South Africa; 3 Center for Astrophysics and Space Astronomy, Department of Astrophysical and Planetary Science, University of Colorado, Boulder, CO 80309 USA; 4 Raman Research Institute, CV Raman Avenue, Bangalore, 560080 India; 5 Tata Institute of Fundamental Research, Homi Bhabha Road, Mumbai, 400005 India; 6 Australia Telescope National Facility, CSIRO Astronomy and Space Science, P.O. Box 76, Epping, NSW 1710 Australia

## Abstract

Electrons at relativistic speeds, diffusing in magnetic fields, cause copious emission at radio frequencies in both clusters of galaxies and radio galaxies through non-thermal radiation emission called synchrotron. However, the total power radiated through this mechanism is ill constrained, as the lower limit of the electron energy distribution, or low–energy cutoffs, for radio emission in galaxy clusters and radio galaxies, have not yet been determined. This lower limit, parametrized by the lower limit of the electron momentum – p_min_ – is critical for estimating the total energetics of non-thermal electrons produced by cluster mergers or injected by radio galaxy jets, which impacts the formation of large–scale structure in the universe, as well as the evolution of local structures inside galaxy clusters. The total pressure due to the relativistic, non–thermal population of electrons can be measured using the Sunyaev-Zel’dovich Effect, and is critically dependent on p_min_, making the measurement of this non–thermal pressure a promising technique to estimate the electron low–energy cutoff. We present here the first unambiguous detection of this Sunyaev-Zel’dovich Effect for a non–thermal population of electrons in a radio galaxy jet/lobe, located at a significant distance away from the center of the Bullet cluster of galaxies.

## Introduction

Galaxy clusters, the largest gravitationally bound structures in the universe, host hot fully ionized plasma – thermal electrons and protons – at temperatures of up to 10–100 million Kelvins. A fraction of galaxy clusters also host energetic population of relativistic electron plasma whose nature is still unknown. Possible sources have been proposed in relation with the existence of intra-cluster shock waves^1^, the injection processes of seed electrons from radio and active galaxies^2^ and dark matter annihilation/decay, e.g.^3,4^.

Once these relativistic electrons are produced by one or a combination of these mechanisms, they diffuse in the magnetized cluster atmosphere and emit non-thermal synchrotron radio emission observed as both extended arches and filamentary structures (named radio relics) or in more homogeneously diffuse halos (named radio halos).

A significant and critical issue in the study of non-thermal electrons through synchrotron emission is the energy distribution of these accelerated particles. Basic parameters that characterize the energy distribution of the electrons in the atmospheres of the galaxy cluster are the high- and low–energy cutoffs.

The energy spectrum of synchrotron emission produced by the accelerated electrons in galaxy clusters is expected to follow a power law whose intensity decreases with increasing energy/frequency: thus, it is the low-energy cutoff that is crucial for determining the total energetics of the cluster-wide radio emission.

Radio emission from jets of radio galaxies is also due to synchrotron emission of relativistic electrons, and therefore also has a power law, and the low-energy cutoff is similarly a critical quantity to determine the energetics of the radio jets associated with these radio galaxies.

Both thermal and non–thermal populations of electrons in galaxy clusters, as well as the mostly non–thermal populations of electrons in the jets of radio galaxies, are energetic enough to cause photons, traveling from the early universe (which constitute the Cosmic Microwave Background or CMB), to shift to higher energies, hence shifting the entire CMB spectrum of photons. This is due to the fundamental mechanisms of inverse Compton scattering (ICS) and is usually referred to as the Sunyaev–Zel’dovich Effect (or SZ Effect)^5–9^ for the up-scattering produced by thermal populations of electrons. Due to the universality of the ICS mechanisms, non-thermal and relativistic electrons in galaxy clusters can also up-scatter the CMB photons by largely increasing their final frequency. This leads to a more general form of the SZ Effect^10^ that we refer to as non-thermal SZE for simplicity.

The SZ Effect probes the integrated pressure (or energy density) of the relative electron population (thermal or non-thermal) in galaxy clusters, along the line of sight – and this property renders it a critical probe of the plasma in these cosmic structures, since it yields information complementary to radio emission from synchrotron. While radio emission from synchrotron provides information about the presence of non-thermal electrons embedded in magnetic fields, the SZ Effect provides a “snapshot” of their pressure profiles; in particular, pressure enhancements, along shocks, radio galaxy jets and other regions in galaxy clusters; especially mergers or collisions of these galaxy clusters.

While thermal SZE has been detected in a number of clusters by now^7,11–13^, and while there have been attempts to detect the non-thermal SZE from giant radio galaxy jets/lobes^14^, only an upper limit on the Compton–y parameter of *y* = 1.04×10^−4^ has been derived at 21 GHz for the giant radio galaxy B1358 + 305^14^. However, these authors found from a differential analysis of the intensity in the 21 GHz maps of this giant radiogalaxy that the main source of the relative fluctuations in these projected maps is the excess atmospheric noise rather than the true non-thermal SZE signal induced by the relativistic electrons in B1358 + 305. Therefore, these instrumental limitations did not allow a detection of the SZE in the direction of this radio galaxy. A further analysis of multifrequency data on the SZE in giant radio galaxy lobes^15^ set further constraints on the minimum momentum of the electrons residing in the radiogalaxy lobes and allowed realistic predictions for its visibility at mm wavelengths with Planck, OLIMPO,and Herschel-SPIRE.

### The ‘Bullet’ cluster of galaxies

A spectacular example of an extremely energetic merger or collision of clusters of galaxies is the ‘Bullet’ cluster (1E0657 – 56), a southern sky object, named due to the eponymous shape of the smaller cluster. This cluster merger provided the most direct evidence for the existence of the so–called Dark Matter^16^, whose spatial distribution was found to be significantly displaced with respect to X–ray emission–this is also one of the most X-ray luminous clusters observed. Other reasons that make this cluster rich in non–equilibrium physics, and one of the most interesting objects to study, are: the existence of a strong radio halo^17,18^ which can be observed up to cm–wavelengths^19^, a bright radio relic^20^ which has been observed up to 10 GHz^20,21^, and the presence of a thermal SZE (references^ 22,23^, and references therein).

We present here the first unambiguous detection of the non–thermal SZ Effect in a radio galaxy jet/lobe, which is ∼800 kpc away from the center of the Bullet cluster. Importantly, we use low-frequency radio data in the range (2.1–9.0) GHz to determine the spectrum of the radio lobe and then fit the ATCA 18 GHz SZE observation with a non-thermal SZE model that is computed in a fully relativistic approach.

Throughout the paper, we use a flat, vacuum–dominated cosmological model with Ω_*m*_ = 0.315, Ω_Λ_ = 0.685 and *H*_0_ = 67.3 km s^−1^ Mpc^−1^.

### The radio galaxy RG01

The Bullet cluster was observed using the Australia Telescope Compact Array (ATCA) at 18 GHz center frequency (16–20 GHz range), using the two most compact arrays, H75 and H168. Details of the observations that are used to image the SZE are provided in a reference^19^.

RG01 is one of the radio galaxies detected in the Bullet cluster field at frequencies 2.1, 5.5, 9.0 and 16–24 GHz in ATCA observations. The radio galaxy RG01–and therefore its radio jet/lobe – is located approximately 180″ or ≈ 800 kpc away from the center of the Bullet cluster. The radio galaxy, and the jet/lobe region, is also approximately 160″, or ≈ 700 kpc away from the nearest diffuse radio halo region which is detected up to 10 GHz^20,21^. In addition, there is no detectable X-ray emission with the Chandra space-borne X-ray observatory in this region. These facts (i.e., the large distance between RG01 and the center of the Bullet cluster, the absence of X-ray emission, the region containing RG01 being far away from the shock front as well as the cold front, and the SZE being in the radio jet/lobe region where synchrotron emission has already been detected at 2.1, 5.5 and 9.0 GHz), imply that the SZE signal we detect can only be of non-thermal origin.

The proximity of the detected non-thermal SZE, as well as its distance from the edge of the cluster (>100″, which corresponds to a distance of ∼0.45 Mpc), suggests that it is likely associated with the jet from the galaxy RG01. Thus far, there do not seem to be any indications of any other merger activity in this region^1^, and the shock deduced from X–ray observations is also ∼ 0.4-0.5 Mpc away from our non–thermal SZ detection^24^.

### The non-thermal SZE in the RG01 lobe

**Figure 1:**
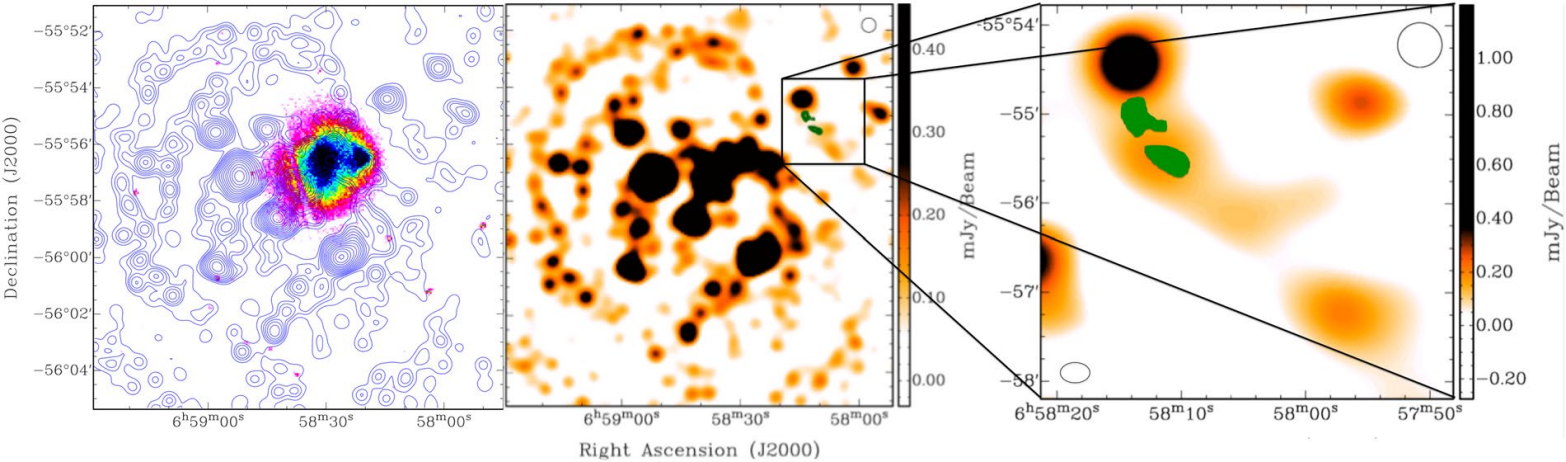
Left: 5.5 GHz contours superposed on X–ray colour plot, showing the relative position of RG01 and the X–ray emission in the Bullet cluster. X–ray data was obtained from the Chandra Data Archive (500 ks observations described in a reference^29^), and was displayed using the KARMA package^28^. Radio contour levels start at 5 σ and increase by a factor of $\sqrt{2}$. The position and relative size of the image in Fig. 1 of a reference^16^ have been indicated and marked. This comparison clearly indicates the significantly larger spread of radio non–thermal emission as compared to the thermal X–ray emission. Middle: The non-thermal SZE detected in the radio galaxy lobe/jet at 18 GHz, displayed as a blue region in the NW region of the 5.5 GHz ATCA image, with 30″ resolution. The SZE region in green is from an image with a resolution of 22″ × 15″. The 5.5 GHz image has a noise rms of 14 *μ* Jy beam^−1^, and the 18 GHz image has a noise rms of 3.5 *μ* Jy beam^−1^. A 30″ beam size is represented in the bottom left corner. Right: A zoom–in to the region marked with a rectangle on the left image; the non-thermal SZE is shown in green, and two distinct regions can clearly be seen, with one closer to the radio galaxy core than the other. The 30″ beam for 5.5 GHz is marked in the top right corner, and the 22″ × 15″ beam for 18 GHz in the bottom left corner. The colour scheme for the 5.5 GHz image is somewhat different, to accentuate small-scale features at 5.5 GHz. To the NW of the radio galaxy and radio lobe/jet there is another radio galaxy, and to the SE is the NW tip of the radio halo at 5.5 GHz, which helps mark the location of the non-thermal SZE in relation to the radio halo.

We detected a non-thermal SZE signal in the jet/lobe of the radio galaxy RG01 located at coordinates (J2000)RA: 06^h^58^m^14.2^s^ DEC: −55°54′25″ and shown in the blue-colored region of Fig. 1. Given the noise rms of 3.5 *μ* Jy beam^−1^, this is a 6.5 σ detection, with the deepest SZE signal being − 22.7 *μ* Jy beam^−1^. We produced images with the five different values of the FWHM, with different weightings, and different amounts of uv–coverages. With these different weighting schemes, as well as different FWHMs of synthesized beams, the size of the two SZE regions turns out to be at most 5% different and also retains the same morphology. Given that the natural and uniform weighing schemes provide different and independent beams, any detection above 3–5 σ that does not depend on the weighing scheme (and therefore on the deconvolution process), can therefore considered as a signal. This demonstrates that our detection of the non-thermal SZE in this region is robust. Additionally, the effect of the synthesized side-lobes is at maximum of 2% at these angular distances away from the brightest sources, assuming that it is 3′ away. Conversion from brightness to temperature units is given by $T=\frac{{\lambda }^{2}}{2k{\rm{\Omega }}}S$ and, given that *λ* = 1.67 cm (i.e., 18 GHz), we obtain *T* = 1.19 × *B* where *B* is in units of $\frac{{\rm{Jy}}}{{{\rm{arcmin}}}^{2}}$. Since the non-thermal SZE we have shown is taken from an image with a beam of 30″ × 30″, our detection of this SZE at −22 *μ* Jy beam^−1^ corresponds, therefore, to −88 *μ* Jy arcmin^−2^; from the formula in the previous equation this yields Δ*T*_*SZ*_ = −105 *μK*, and a Compton-y parameter of $y=-\frac{1}{2}\frac{{\rm{\Delta }}{T}_{{\rm{SZ}}}}{{T}_{{\rm{CMB}}}}=1.9\times {10}^{-5}$.

We note that this value of the Compton parameter is a factor ∼18 times smaller than the upper limit on the SZE signal derived by^14^ in the jets of a giant radio galaxy; this indicates that high sensitivity radio observations are needed to detect such SZE signal.

### Modeling the non–thermal SZ Effect

We model the non-thermal SZE signal we detected in the RG01 lobe using a full relativistic formalism^8^, where the SZE spectrum is given by the expression Δ*I*(*x*) = *I*(*x*) − *I*_0_(*x*). The incoming radiation spectrum is the CMB spectrum ${I}_{0}(x)=2\frac{{(k{T}_{0})}^{3}}{{(hc)}^{2}}\frac{{x}^{3}}{{e}^{x}-1}$, with *x* = *hv*/*kT*_0_ and where *T*_0_ is the CMB temperature today. The resulting SZE spectrum is calculated according to the equation $I(x)={\int }_{-\infty }^{+\infty }{I}_{0}(x{e}^{-s})P(s)ds$, where *P*(*s*) is the photon redistribution function (yielding the probability of a logarithmic shift *s* = In(*v*′/*v*) in the photon frequency due to the inverse Compton scattering process) that depends on the electron spectrum producing the CMB Comptonization, and includes all the relativistic corrections. It is calculated by the sum of the probability functions to have *n* scatterings, *P*_*n*_(*s*), weighted by the corresponding Poissonian probability: $P(s)={\sum }_{n=0}^{+\infty }\frac{{e}^{-\tau }{\tau }^{n}}{n!}{P}_{n}(s)$, where the optical depth *τ* is given by the integral along the line of sight $\ell$ of the electron density $\tau ={\sigma }_{T}\int {n}_{e}d\ell$, where *n*_*e*_ is the electron plasma density. Each function *P*_*n*_(*s*) is given by the convolution product of *n* single scattering probability functions *P*_1_(*s*):

$${P}_{n}(s)=\mathop{\underbrace{{P}_{1}(s)\otimes \ldots \otimes {P}_{1}(s)}}\limits_{\,{\rm{n}}\,{\rm{times}}\,}{\rm{where}}\,{P}_{1}(s)={\int }_{0}^{\infty }{f}_{e}(p){P}_{s}(s,p)dp,$$

and where *f*_*e*_(*p*) is the electron momentum distribution function (normalized as to have ${\int }_{0}^{\infty }{f}_{e}(p)dp=1$), and *P*_*s*_(*s*, *p*) is the function that gives the probability to have a frequency shift *s* by an electron with a-dimensional momentum p = *βγ*, and is given by the physics of the inverse Compton scattering process (see, e.g. two references^8,25^).

For non-thermal electrons we use a single power-law electrons momentum distribution with a minimum momentum *p*_1_

$${f}_{e}(p)\propto {p}^{-s{}_{e}};{p}_{1}\le p\le {p}_{2},$$

and we assume a high value of the maximum momentum (*p*_2_ = 10^8^).

Our 18 GHz observation for the RG01 SZE signal can be fitted, in principle, with both a thermal or a non-thermal electron population with different values of the spectral index and the minimum momentum of electrons (for the non-thermal SZE) and of the temperature (for the thermal SZE), leaving the optical depth as a free parameter: this is, in fact, the result of a degeneracy in the SZE parameters at low-frequencies. However, the absence of any detectable X–ray emission in this region, its large distance from the centre of the cluster merger (≈ 800 kpc), and the absence of any diffuse emission in a region roughly 400 kpc region E to W from the westernmost edge of X–ray emission, imply that emission observed in this region does not have a thermal origin.

In order to break this parameter degeneracy, we obtained information about the spectral index of the electrons in the RG01 lobe from the observed radio spectrum in the frequency range 2.1–9.0 GHz: the average radio spectral index measured between 2.1 and 9 GHz is *α*_*r*_ = 1.1 ± 0.15. This corresponds to a range of electrons spectral index values in the range 2.9 ≤ *s*_*e*_ ≤ 3.5, where *s*_*e*_ = 2*α*_*r*_ + 1. The shape of the radio spectrum indicates that we are in the presence of a quite typical non-thermal electron distribution in the RG01 lobe; this important fact allows us to constrain the range of possible SZE models that can fit the observed SZE signal at 18 GHz, thus restricting our analysis to non-thermal models of the SZE.

**Figure 2:**
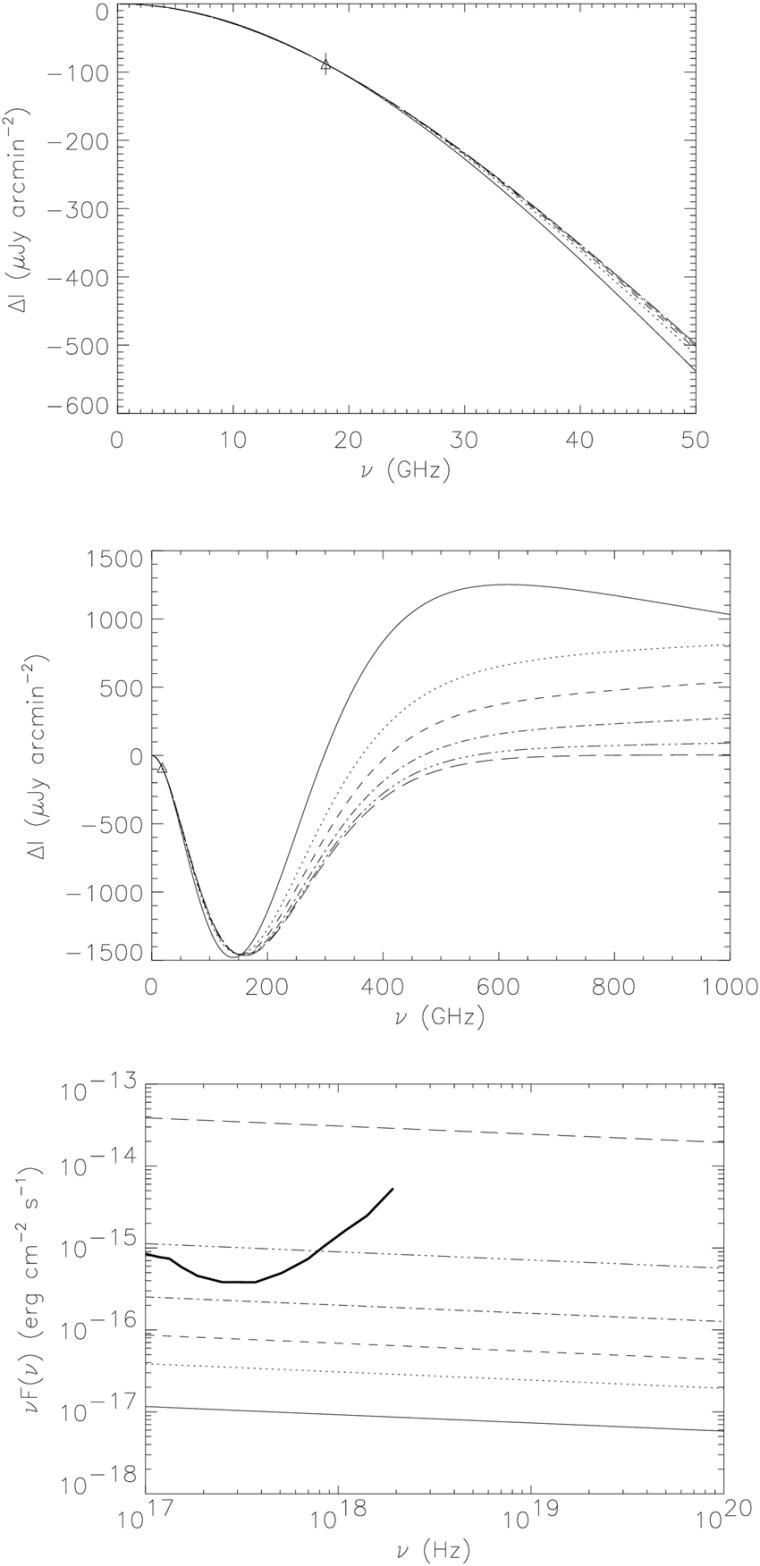
Spectrum of the non-thermal SZE (in surface brightness units) at low (upper panel) and high frequencies (middle panel) and of the ICS emission in the X-rays integrated inside a square with side length of 4 arcsec (lower panel) for an electron non-thermal population with *s*_*e*_ = 3.2. The following models are plotted: solid line: non-thermal SZE with minimum momentum of electrons *p*_1_ = 1 and optical depth *τ* = 7.6 × 10^−5^; dotted line: *p*_1_ = 2 and *τ* = 5.5 × 10^−5^; dashed line: *p*_1_ = 3 and *τ* = 5.1 × 10^−5^; dot-dashed line: *p*_1_ = 5 and *τ* = 4.8 × 10^−5^; three dots-dashed line: *p*_1_ = 10 and *τ* = 4.7 × 10^−5^; long-dashed line: *p*_1_ = 50 and *τ* = 4.6 × 10^−5^. The sensitivity of Chandra is plotted for an integration time of 100 ks.

Figure 2 reports the non-thermal SZE in the RG01 lobe calculated with the value of the average radio spectral index measured between 2.1 and 9 GHz *α*_*r*_ = 1.1 corresponding to *s*_*e*_ = 3.2 and is shown at low frequencies (*v* < 50 GHz) and at high frequencies (*v* < 1000 GHz); in addition, we show in this figure the ICS X-ray emission expected for several values of the minimum momentum *p*_1_ of the non-thermal electron distribution. All the non-thermal SZE models which are consistent with the observed radio spectrum of the RG01 lobe can fit the ATCA SZE signal at 18 GHz confirming that the SZE signal we detected is of non-thermal origin and it is produced by a non-thermal electron population whose energy spectrum is consistent with the observed radio synchrotron spectrum.

This is the first detection of a non-thermal SZE effect in the lobe of a radio galaxy. The upper limit on the X-ray emission from the RG01 lobes provided by Chandra indicates an upper limit on the value of *p*_1_ between 5 and 10 that correspond to minimum electron energies *E*_min_ < 2.5 − 5 MeV.

The central panel of Fig. 2 shows that a more precise determination of the value of *p*_1_ can be obtained by measuring the spectrum of the non-thermal SZE at high frequencies, i.e. at the crossover frequency, that can vary between ∼250 and ∼500 GHz depending on the value of *p*_1_, or in the high energy part of the spectrum, where the SZE is positive. This is in agreement with previous studies of the properties of non-thermal electrons in radio galaxy lobes from SZE measurements^26^. We note that a measurement of *p*_1_ can lead to an estimate of the optical depth *τ*, and this value in turn leads to crucial information about the physics of radio galaxy lobes; namely, the density of non-thermal electrons and the intensity of the magnetic field in the lobe^26^.

## Conclusions

The first detection of the non-thermal SZE presented here in a radio galaxy jet, after its first theoretical prediction^27^ is a significant step towards the characterization of the low–energy cut–off in a non–thermal plasma, and its usefulness and importance therefore cuts across several fields in astrophysics. Extension of the non-thermal SZE observed in the lobe of RG01 at higher frequencies can also be observed with mm and sub-mm experiments with appropriate sensitivity and angular resolution (like ALMA and Millimetron).

Future detection of X-ray emission from the same physical process, i.e. the up-scattering of CMB photons by non–thermal populations of electrons in the lobe of RG01, combined with radio and SZ Effect, will provide a value of the overall energy extension and spectral shape of the energy spectrum of these non-thermal electrons residing in lobes of radio galaxies.
